# Synthesis, Characterization, and Electrochemical Behavior of LiMn_x_Fe_(1−x)_PO_4_ Composites Obtained from Phenylphosphonate-Based Organic-Inorganic Hybrids

**DOI:** 10.3390/ma11010056

**Published:** 2017-12-30

**Authors:** Alessandro Dell’Era, Mauro Pasquali, Elvira Maria Bauer, Stefano Vecchio Ciprioti, Francesca A. Scaramuzzo, Carla Lupi

**Affiliations:** 1 Department of Basic and Applied Sciences for Engineering (SBAI), Sapienza University of Rome, Via del Castro Laurenziano 7, 00161 Rome, Italy; mauro.pasquali@uniroma1.it (M.P.); francesca.scaramuzzo@uniroma1.it (F.A.S.); 2 Istituto di Struttura della Materia ISM—CNR, Via Salaria, km. 29.300, C.P. 10, 00015 Rome, Italy; elvira.bauer@ism.cnr.it; 3 Department Chemical Engineering Materials Environment DICMA, University Sapienza Rome, Via Eudossiana 18, 00184 Rome, Italy; carla.lupi@uniroma1.it

**Keywords:** lithium-ion battery, LiMn_x_Fe_(1−x)_PO_4_, carbon coating, pseudo-diffusion coefficient, potential step voltammetry, electrochemical impedance spectroscopy

## Abstract

The synthesis of organic-inorganic hybrid compounds based on phenylphosphonate and their use as precursors to form LiMn_x_Fe_(1−x)_PO_4_ composites containing carbonaceous substances with sub-micrometric morphology are presented. The experimental procedure includes the preliminary synthesis of Fe^2+^ and/or Mn^2+^ phenylphosphonates with the general formula Fe_(1−x)_Mn_x_[(C_6_H_5_PO_3_)(H_2_O)] (with 0 < x < 1), which are then mixed at different molar ratios with lithium carbonate. In this way the carbon, obtained from in situ partial oxidation of the precursor organic part, coats the LiMn_x_Fe_(1−x)_PO_4_ particles. After a structural and morphological characterization, the electrochemical behavior of lithium iron manganese phosphates has been compared to the one of pristine LiFePO_4_ and LiMnPO_4_, in order to evaluate the doping influence on the material.

## 1. Introduction

Nowadays, lithium-ion batteries are the most developed energy sources for modern portable electronics and their use in automotive application is also increasing [1,2,3,4,5,6,7,8]. So far, several materials, such as LiCoO_2_ and LiMn_2_O_4_, have been used as cathode, but recently LiFePO_4_ has attracted researchers’ interest due to its high specific energy, which may reach 580 Wh/kg, and relatively low production cost [9,10,11,12,13,14]. As a drawback, LiFePO_4_ has low ionic diffusivity and conductibility [15,16,17], which limits its use as cathode. The electronic conductibility of LiFePO_4_ can be enhanced by using several materials processing methods such as in situ carbon synthesis, or by particle coating with conductive carbons [18], or by an ion doping approach [19,20,21,22,23,24]. In the latter case, the oxidized form of LiFePO_4_ should be modified with cations having ionic radius slightly higher than Fe^2+^ and Fe^3+^, such as manganese, facilitating a wider channel for lithium-ion diffusion, increasing the mobility of lithium ion but, at the same time, avoiding the structure to be stressed [25]. Moreover, the LiFePO_4_ particles size should be reduced to decrease the average free lithium pathway in insertion/de-insertion process and raise the performances. Indeed, in this way, all the material can be effectively used, consequently enhancing the specific capacity. The aim of this work has been to analyze the electrochemical performance of lithium iron phosphate with the addition of manganese LiMn_x_Fe_(1−x)_PO_4_ starting from Fe^2+^ and Mn^2+^ phenylphosphonates (general formula Fe[(C_6_H_5_PO_3_)(H_2_O)] or Mn[(C_6_H_5_PO_3_)(H_2_O)]) in appropriate ratio as metal-organic precursor and Li_2_CO_3_ as inorganic precursor. Moreover, in order to verify possible differences in the electrochemical performances, one of the lithium iron manganese phosphates, i.e., LiFe_0.9_Mn_0.1_PO_4_, has been also synthesized by using the precursor Fe_0.9_Mn_0.1_[(C_6_H_5_PO_3_)(H_2_O)]. Thermal, structural, and morphological analyses have been performed on both precursors and final materials; finally, an electrochemical characterization has been carried out on all prepared samples to evaluate if the synthesis process and the hetero-metal adding degree can influence their specific capacity.

## 2. Materials and Methods

### 2.1. Synthesis of Precursors

Analytical grade (Sigma Aldrich Chemical Co., Darmstadt, Germany) phenylphosphonic acid (H_2_C_6_H_5_PO_3_), ammonium hydroxide (NH_4_OH), iron(II) sulphateheptahydrate (FeSO_4_·7H_2_O), and manganese(II) sulphatemonohydrate (MnSO_4_·H_2_O) were used for the synthesis without further purification. HPLC (High Pressure Liquid Chromatography) water (Carlo Erba, Milan, Italy) was used as a solvent. Usual Schlenck techniques were used to prepare the phenylphosphonate precursor materials.

All metal(II) phosphonate precursors, i.e., Fe_0.9_Mn_0.1_(C_6_H_5_PO_3_)(H_2_O) (**P1**), Fe[(C_6_H_5_PO_3_)H_2_O] (**P2**) and Mn(C_6_H_5_PO_3_)(H_2_O) (**P3**),were obtained following the synthetic procedure described previously for iron(II) phenylphosphonate monohydrate Fe[(C_6_H_5_PO_3_)H_2_O] [21,22]: 10 g (63.25 mmol) of H_2_C_6_H_5_PO_3_ were suspended under continuous stirring in 50 mL of water in a 100 mL two-necked flask (flask 1). NH_4_OH (about 9.5 mL, 30% in H_2_O) was added, up to pH = 7, to the white colloidal suspension obtained, thus giving the water soluble ammonium salt of the phenylphosphonic acid (NH_4_)_2_(C_6_H_5_PO_3_). In another 100 mL two-necked flask (flask 2), 7 g (25.17 mmol) of FeSO_4_·7H_2_O were dissolved in 35 mL of degassed water. After the complete dissolution of ferrous sulphate, the degassed aqueous solution of (NH_4_)_2_(C_6_H_5_PO_3_) was transferred from flask 1 to flask 2 under a stream of inert gas and with a filtration system. The filtration system guarantees the transfer of a filtered and clear solution of the ammonium salt of phenylphosphonic acid to iron(II) sulphate. During the transfer, iron(II) phenylphosphonate, Fe[(C_6_H_5_PO_3_)H_2_O] formed instantaneously as a white flaked precipitate. The white colloidal suspension thus obtained was maintained under continuous stirring under flowing nitrogen for approx. 2 h (pH_fin_ = 6.14). The precipitate was then filtered in air, washed with water to neutrality with acetone, and finally air-dried. Three different metal(II) phenylphosphonate precursors, reported in Table 1, were isolated by the former preparation method.

### 2.2. Synthesis of LiMn_0.1_Fe_0.9_PO_4_

In particular, LiMn_0.1_Fe_0.9_PO_4_ was synthesized starting from different precursors following different procedures, and the final products obtained were compared in terms of morphology and electrochemical performances. In detail, LiMn_0.1_Fe_0.9_PO_4_ was synthesized by mixing in a mechanical mill Li_2_CO_3_ (analytical grade by Sigma Aldrich Chemical Co.) and either the precursor **P1** Fe_0.9_Mn_0.1_(C_6_H_5_PO_3_)·H_2_O (sample **S1**) or the precursors **P2** Fe(C_6_H_5_PO_3_)·H_2_O and **P3** Mn(C_6_H_5_PO_3_)·H_2_O in molar ratio 0.9/0.1 (sample **S2**). After the grinding process the light-grey powder homogeneous mixture of the reagents was placed in an alumina crucible and transferred into the central zone of a tubular furnace for calcination. In order to maintain an inert environment, the mixture of reagents was degassed for 1 h at room temperature under nitrogen flowing. Successively the powder underwent a calcination at 600 °C for 16 h under nitrogen flowing. The calcined product was then cooled under inert gas to room temperature, thus obtaining a fine black powder.

### 2.3. Synthesis of LiMn_x_Fe_(1−x)_PO_4_ (with x = 0.05, 0.1, 0.5, 0.9, and 0.95)

The synthesis was performed starting from Li_2_CO_3_, **P2** and **P3** precursors, as described in the previous paragraph. In order to obtain all the desired compounds, **P2** and **P3** were mechanically mixed with a molar ratio (1−x):x respectively, where x = 0.05, 0.1, 0.5, 0.9, and 0.95, followed by thermal treatment under inert atmosphere as reported above.

## 3. Results and Discussion

### 3.1. Precursors Characterization

**Figure 1:**
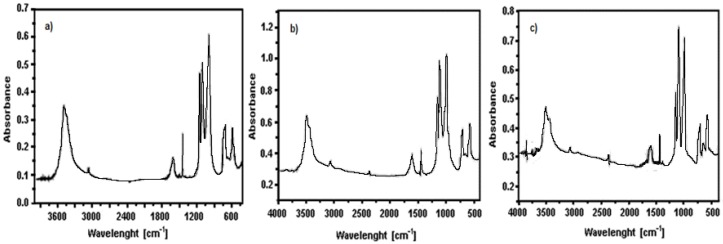
FT-IR spectra of precursors: (**a**) Fe_0.9_Mn_0.1_(C_6_H_5_PO_3_)(H_2_O) (**P1**); and (**b**) Fe[(C_6_H_5_PO_3_)H_2_O] (**P2**) and (**c**) Mn(C_6_H_5_PO_3_)(H_2_O) (**P3**).

Infrared spectra of the different monohydrate precursors appear quite similar and present several bands, as shown in Figure 1. The bands between 3420 and 3470 cm^−1^ and the band at 1604 cm^−1^ correspond respectively to the stretching and bending vibrations of the water molecule of M(II) phenylphosphonate monohydrate (M = Fe, Mn, Fe_0.9_Mn_0.1_). Other characteristic bands of this compounds are located between 3074 and 3054 cm^−1^ and are associated with the stretching vibrations of the C-H bond of the phenyl group, while the band at 1438 cm^−1^ corresponds to C-C bond stretching of the same group. Finally, in the region between 1200–970 cm^−1^, the characteristic stretching vibrations of the P-O bond of the anion (PO_3_)^2−^ are observed. The complete conversion of phenylphosphonic acid to metal(II) phosphonate is confirmed by the absence of the typical OH-binding strain vibrations of the P-OH group, generally observed as wide bands between 2900 and 2300 cm^−1^.

**Figure 2:**
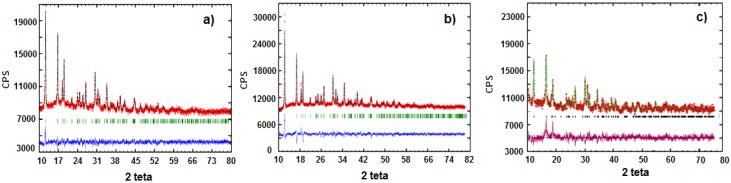
X-ray Diffraction pattern of precursors: (**a**) Fe_0.9_Mn_0.1_(C_6_H_5_PO_3_)(H_2_O) (**P1**); and (**b**) Fe[(C_6_H_5_PO_3_)H_2_O] (**P2**) and (**c**) Mn(C_6_H_5_PO_3_)(H_2_O) (**P3**).

In Figure 2, the X-ray diffractograms and the refining results obtained by the Rietveld method are reported. The only crystalline phase present in the analyzed precursor powders corresponds to the expected M(II)phenylphosphonate (M = Fe, Mn, Fe_0.9_Mn_0.1_) [26,27]. All the three phenylphosphonate precursors crystallize in the orthorhombic spatial group Pmn2_1_. In Table 2 the cell parameters for the different samples are reported. Excluding lattice parameter “b”, it is possible to state that passing from iron to manganese produces an increasing of the cell size.

### 3.2. Characterization of LiFe_0.9_Mn_0.1_PO_4_

**Figure 3:**
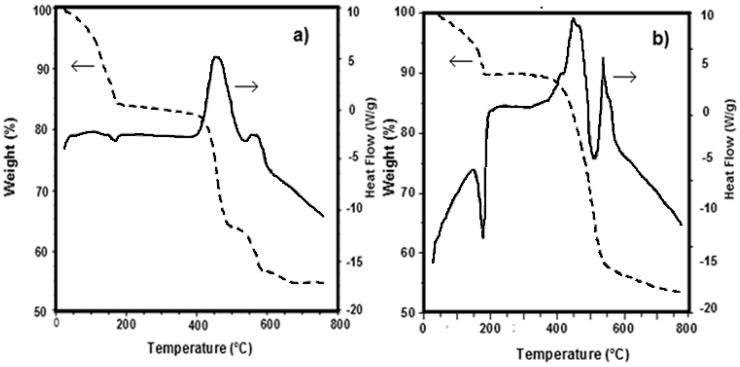
DSC-TG curves of: (**a**) sample **S1**: **P1**/Li_2_CO_3_; and (**b**) sample **S2**: (**P2**:**P3** = 0.9:0.1)/Li_2_CO_3_.

DSC-TG (Differential Scanning Calorimetry–Thermo-Gravimetry) curves obtained under nitrogen flow for Fe_0.9_Mn_0.1_(C_6_H_5_PO_3_)·H_2_O (**P1**)/Li_2_CO_3_ mixture (sample **S1**) and for Fe(C_6_H_5_PO_3_)·H_2_O (**P2**) and Mn(C_6_H_5_PO_3_)·H_2_O (**P3**) (**P2**:**P3** = 0.9:0.1)/Li_2_CO_3_ mixture (sample **S2**) are reported, respectively, in Figure 3a,b. The thermal behaviour of both mixtures resulted to be quite similar.

Part of the water, probably physically adsorbed on the sample, is lost at temperatures lower than 100 °C. The remaining part, i.e., the crystallization water, is lost at about 200 °C: this phenomenon is clearly highlighted by the end thermal peak observable in the DSC curves at 180 °C. The weight loss up to 180 °C is about 11–15%. At higher temperatures two exothermic effects are displayed in the DSC curves, namely at 400 and 550 °C, which are accompanied by a weight loss in the TG curves of about 25–30% and 8–10%, respectively.

**Figure 4:**
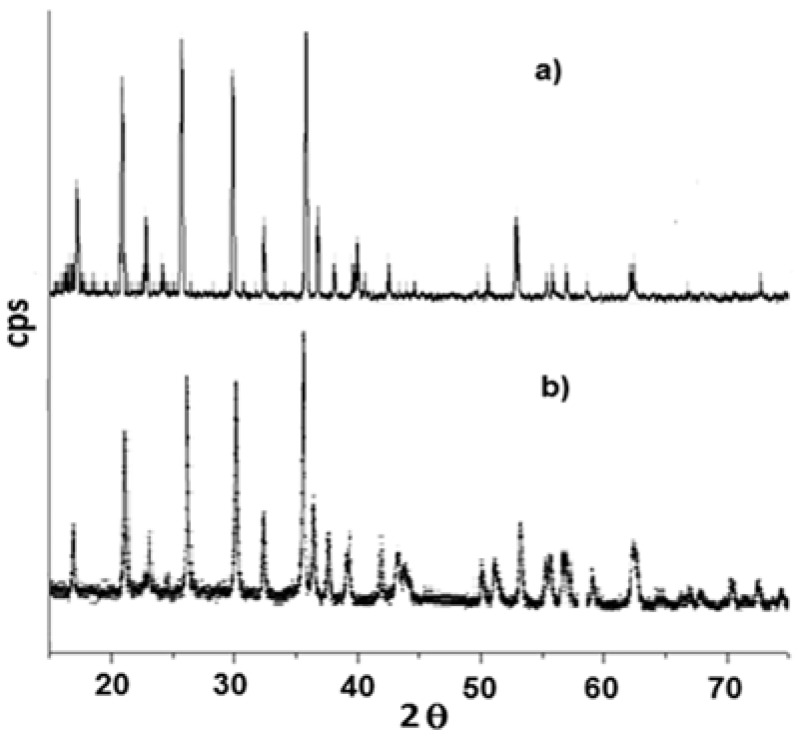
X-ray Diffraction pattern of LiFe_0.9_Mn_0.1_PO_4_: (**a**) sample **S1**: **P1/**Li_2_CO_3_; and (**b**) sample **S2**: (**P2**:**P3** = 0.9:0.1)/Li_2_CO_3_.

**Figure 5:**
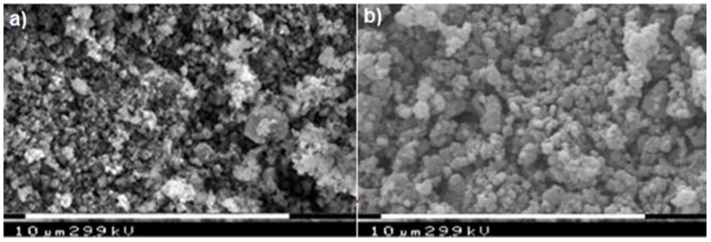
SEM images of LiFe_0.9_Mn_0.1_PO_4_: (**a**) sample **S1**: **P1/**Li_2_CO_3_; and (**b**) sample **S2**: (**P2**:**P3** = 0.9:0.1)/Li_2_CO_3_.

**Figure 6:**
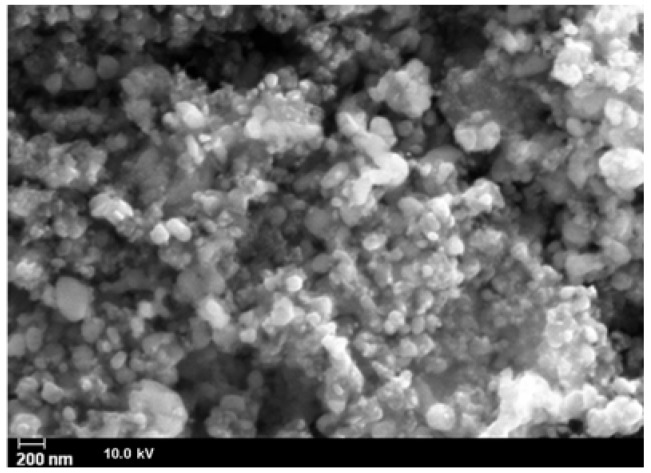
Morphology and size details at higher magnification.

These effects are related to the decomposition of carbonate and organo-phosphonates and to the formation of lithium metal(II) phosphate. Such experimental evidences are in good agreement with literature, according with the dehydration of some hydrate metal phosphates which proceeds by both anion disproportion and condensation. The X-ray powder diffraction patterns of LiMn_0.1_Fe_0.9_PO_4_ prepared from either **P1** and Li_2_CO_3_ or **P2**, **P3**, and Li_2_CO_3_ precursors are very alike as well, as shown in Figure 4. Both belong to orthorhombic space group *Pnma* (olivine like structure) [13]. The similarity between these two samples is evident also from SEM (Scanning Electron Microscopy) images reported in Figure 5 and Figure 6.

The particles show a comparable morphology. In both samples the particles appear agglomerated and the presence of two phases can be noted: the former, likely carbon, is characterized by very small spheres, while the second one (LiMn_0.1_Fe_0.9_PO_4_) is characterized by larger and less regular particles.

The particles are spheroidal, or in any case there is no dimension that prevails over the others during the growth, like for example in a needle structure; such experimental evidence suggests that during the thermal treatment strong nucleation with the formation of small nucleuses growing indifferently in all directions occurs. In Figure 6 a higher magnification highlights the formation of very small particles with nanometric size. Actually, the formation of carbon on the active material surface can inhibit the particle grow ensuring a tiny granulometry and possibly can provide good conductibility and electric contact between particles [13,14,15,16,17,18,19,20,21,22,23,24,25,26,27,28].

**Figure 7:**
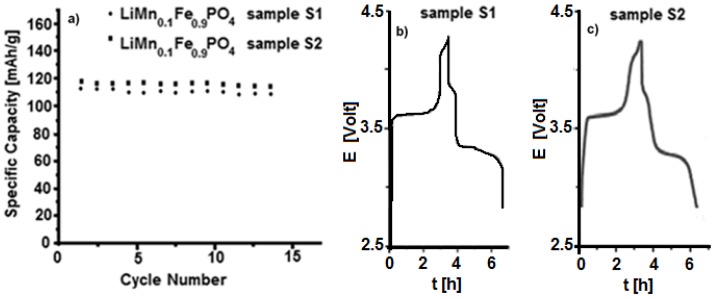
(**a**) Electrochemical galvanostatic tests at C/5 rate for samples **S1** and **S2**; and (**b**) voltage profile of sample **S1**; (**c**) voltage profile of Sample **S2**.

Electrochemical galvanostatic tests on both samples **S1** and **S2** are shown in Figure 7. The cathode electrodes have been charged and discharged with a current value of C/5 and a specific capacity of about 115–120 mAh/g has been obtained. The materials seem to show similar behaviour, even though sample **S2**, obtained by using **P2**, **P3** and Li_2_CO_3_ precursors, presents higher capacity and seems to be more stable upon cycling. On the other hand, the synthesis by **P1** precursor always produces a less performing material, even though it is not straightforward to give an explanation for such different behaviour. Several tests have been performed for each material, and the results are well reproducible.

### 3.3. Characterization of LiMn_x_Fe_(1−x)_PO_4_ (with x = 0, 0.05, 0.1, 0.5, 0.9, 0.95, and 1)

Furthermore, taking in consideration this electrochemical results, a series of LiMn_x_Fe_(1−x)_PO_4_ (with x = 0, 0.05, 0.1, 0.5, 0.9, 0.95 and 1), obtained only from **P2** and/or **P3** and Li_2_CO_3_ precursors, have been prepared and characterized.

**Figure 8:**
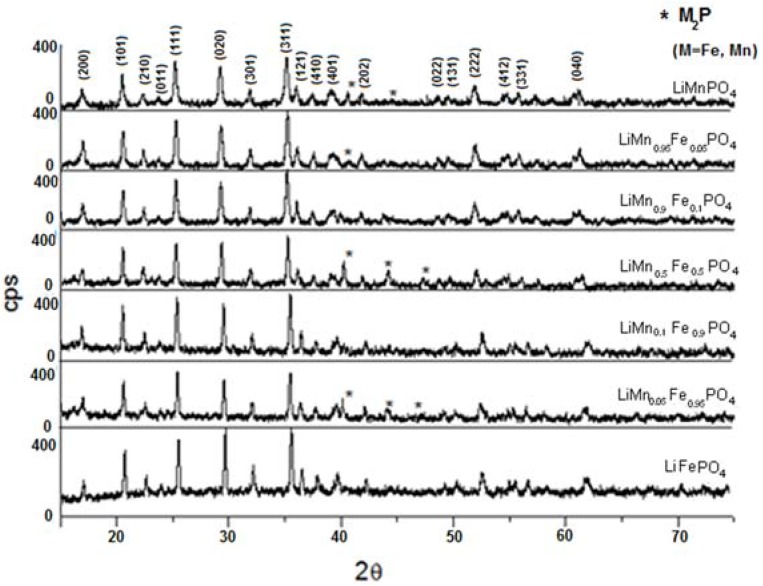
X-ray powder diffraction pattern of LiMn_x_Fe_(1−x)_PO_4_ (x = 0; 0.05; 0.1; 0.5; 0.9; 0.95; 1).

The powder X-ray diffraction spectra of LiMn_x_Fe_(1−x)_PO_4_ (x = 0; 0.05; 0.1; 0.5; 0.9; 0.95; 1) are shown in Figure 8. As it can be observed, substituting manganese in lithium manganese phosphate with iron(II) slightly moves all peaks to the right, although the similarity of the crystalline structure of the two lithium metal(II) phosphates is clear.

Moreover, in Table 3 the refinement results for cell parameters and crystallites size T, calculated by Scherrer equation (T = 0.9λ/Δ(2θ) cosθ), have been reported and it is clear that passing from LiFePO_4_ to LiMnPO_4_ the cell size slightly increases, while the crystallites size decreases.

**Figure 9:**
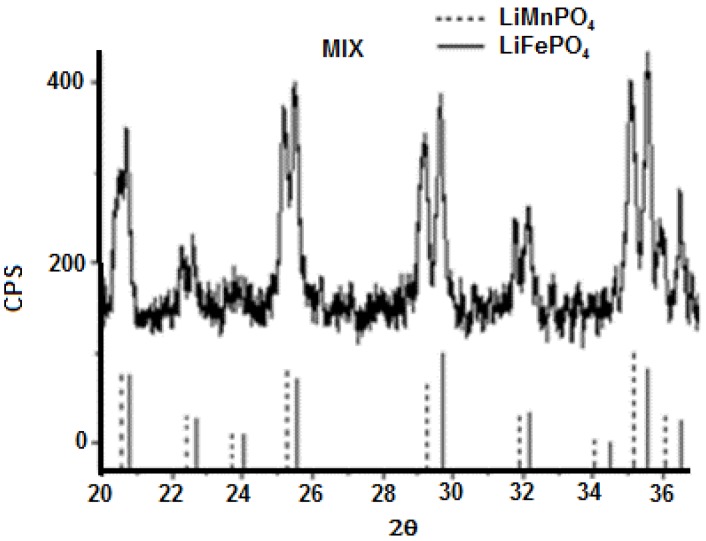
Comparison between X-ray Diffraction spectra of a binary mixture of LiFePO_4_ and LiMnPO_4_ and corresponding pure lithium metal phosphates.

In some samples (x = 0.05; 0.5; 0.95; 1) the presence of iron phosphide (Fe_2_P) impurities have been detected. Replacement of the bivalent hetero-metal atom does not affect the crystalline structure of pure lithium iron and manganese phosphate. Indeed the crystalline structure (space group) is the same but substitution of iron with manganese (different atomic radius) in effect shifts the peak positions slightly and this is visible also in the reported XRD spectra. What is important here is that for all samples one, unique crystalline phase has been detected while the mechanic mixture clearly shows peak splitting due to the presence of two crystalline phases. On the other hand, when simply mixing together (0.5:0.5) LiMnPO_4_ and LiFePO_4_, the observed X-ray diffractogram shows a splitting of the peaks, as reported in Figure 9. Actually, in this case two similar crystalline structures presenting slightly different peak positions are present, therefore, two distinct phases and a splitting of peaks are evident. It is worth to note that in the case of manganese-iron phosphate synthesized from metal(II) phenylphosphonate mixtures as described before, even when Mn(II) and Fe(II) are present in equal ratio, as in LiMn_0.5_Fe_0.5_PO_4_, formation of only one crystalline phase has been observed.

The results of BET analysis for cathodic powders obtained are reported in Table 4 along with the carbon weight percentage determined by elemental analysis, which ranges from 10% to 13.7%. This percentage value has been also confirmed by EDX analysis performed on some samples. The average specific surface is equal to about 115 m^2^·g^−1^.

**Figure 10:**
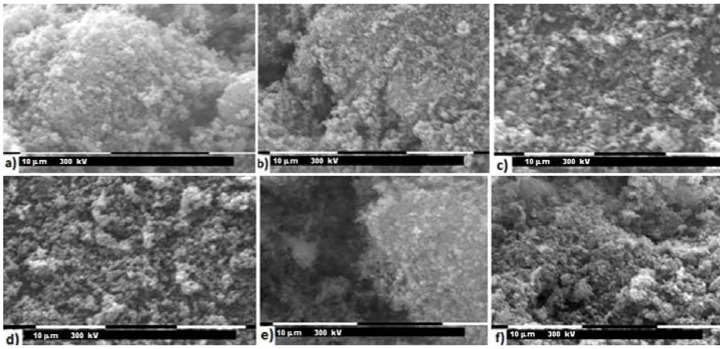
SEM images of: (**a**) LiFePO_4_; (**b**) LiMn_0.05_Fe_0.95_PO_4_; (**c**) LiMn_0.5_Fe_0.5_PO_4_; (**d**) LiMn_0.9_Fe_0.1_PO_4_; (**e**) LiMn_0.95_Fe_0.05_PO_4_; and (**f**) LiMnPO_4_.

The SEM images in Figure 10 show similarity of both morphology and particle size of the various samples. Indeed, identical considerations already done for LiMn_0.1_Fe_0.9_PO_4_ and no particular differences can be highlighted.

The electrochemical tests indicate that pure lithium iron phosphate is the material with the highest specific capacity, i.e., about 150 mAh·g^−1^. Upon increasing of manganese content, the capacity gradually drops, reaching the significantly low value of 23 mAh·g^−1^ for LiMn_0.95_Fe_0.05_PO_4_. Therefore, regarding the specific capacity, the presence of manganese does not seem to have particular advantages.

**Figure 11:**
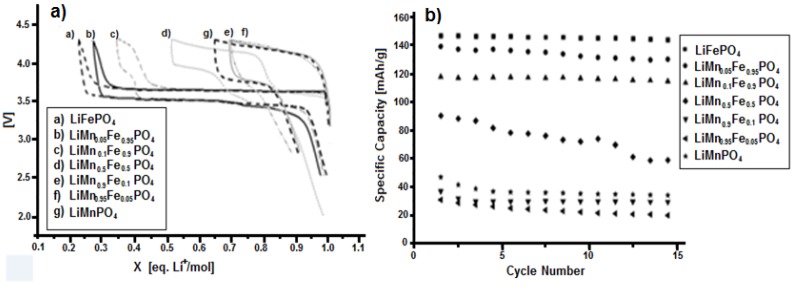
Results of tests at C/5 for LiMn_x_Fe_(1−x)_PO_4_ (x = 0.05; 0.1; 0.5; 0.9; 0.95): (**a**) Charge-discharge curves; and (**b**) specific capacity.

As it can be seen from Figure 11a, the only advantage shown by the presence of manganese is a higher insertion-deinsertion potential value. In fact, the potential value of the Mn^3+^/Mn^2+^ redox couple is 4.15 V, while for the Fe^3+^/Fe^2+^ redox couple it results to be 3.5 V, both vs. Li^0^/Li^+^.

The LiMn_0.5_Fe_0.5_PO_4_ compound exhibits a poorly stability upon cycling, while the compounds with a higher percentage of manganese show a low capacities. Taking into consideration the two pure compounds, namely LiFePO_4_ and LiMnPO_4_, they both have capacity higher than the respective modified compounds. In particular, the presence of Mn(II) decreases the capacity of LiFePO_4_ more than the substituition of manganese with iron in LiMnPO_4_. Indeed the plateaus at 3.5 V for LiMn_0.9_Fe_0.1_PO_4_ and LiMn_0.95_Fe_0.05_PO_4_ are absent, while for LiMn_0.5_Fe_0.5_PO_4_ the plateau at 3.5 V is shorter than the one at 4.15 V. Even if there is just one phase, as it is possible to note by XRD, lithium ion insertion into the structure induces either reduction of Fe^+3^ to Fe^+2^ at about 3.5 V or the reduction of Mn^+3^ to Mn^+2^ at about 4.1 V, producing in both cases an equilibrium between the oxidized and reduced form (namely, MePO_4_/LiMePO_4_, where Me is Fe or Mn), and then determining the stress inside the structure. Such stress, produced by hetero-atom reduction, is even enhanced when its amount is very low, since, in this case, around its position dissimilar atoms are present and the redox reaction could be inhibited. However, the presence of low quantity of manganese can help the lithium insertion into the iron-base structure, but not vice versa.

Among the different compounds of the series, only those of lithium iron-phosphate with low manganese content have been taken into account for further investigation, since they have the highest capacities. To evaluate the performances of those samples, the reversibility degree of the insertion-deinsertion process, the pseudo-diffusion lithium coefficient and the charge transfer resistance, were calculate by PSV, PITT, and EIS experiments, respectively.

**Figure 12:**
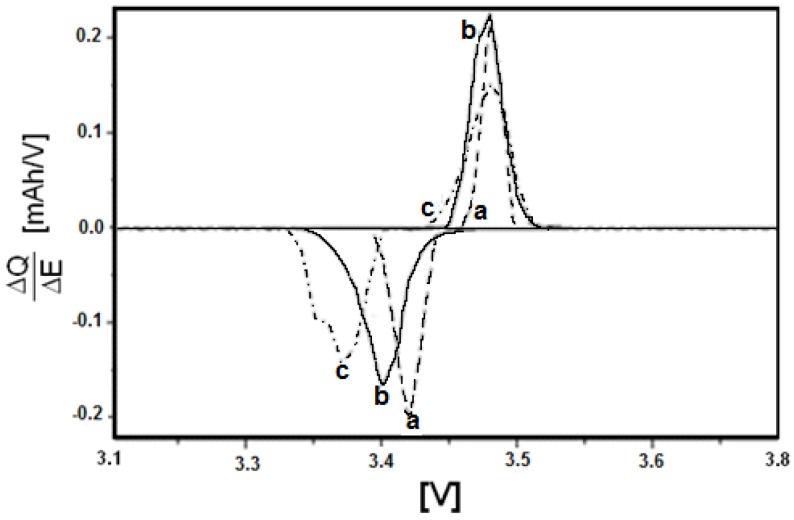
Potential spectroscopy of: (**a**) LiMn_0.1_Fe_0.9_PO_4_; (**b**) LiMn_0.05_Fe_0.95_PO_4_; and (**c**) LiFePO_4_.

In Figure 12 potential step voltammetry is shown for LiFePO_4_, LiMn_0.05_Fe_0.95_PO_4_, and LiMn_0.1_Fe_0.9_PO_4_. In this picture, the insertion-deinsertion process can be observed in correspondence of two peaks. The upward peaks correspond to the oxidation process at approximately 3.47 V for all samples (odd sweeps), while the downward peaks represent the reduction process at approximately 3.37, 3.40, and 3.42 V (even sweeps) for LiFePO_4_, LiMn_0.05_Fe_0.95_PO_4_, and LiMn_0.1_Fe_0.9_PO_4_, respectively. In general terms, the shorter the distance between oxidation and reduction peaks, the higher the reversibility of the process. In the present case it is possible to recognize that manganese content in LiFePO_4_ increases the process reversibility. The average value of pseudo-diffusion coefficient has been also evaluated determining the Cottrell region for the potential step voltammetry corresponding to the deinsertion process, by using the PITT technique [29,30,31], and assuming the average particle radius as the diffusion characteristic length, L, equal to about 0.1 × 10^−4^ cm. Indeed, as said before, the insertion of lithium takes place by means of several reaction fronts, and a pseudo-diffusivity coefficient should be more correctly defined, despite McKinnon and Hearing’s assumption [32], who found that it is not possible to distinguish between two different diffusion models based on continuous (solid solution formation) or not continuous (two-phase formation) charging procedures. In Table 5 the pseudo-diffusion coefficient value for both pure LiFePO_4_ and the materials with low manganese content has been reported. Increasing the manganese content, the value enhances very slightly, so that only few changes can be reached with manganese adding.

**Figure 13:**
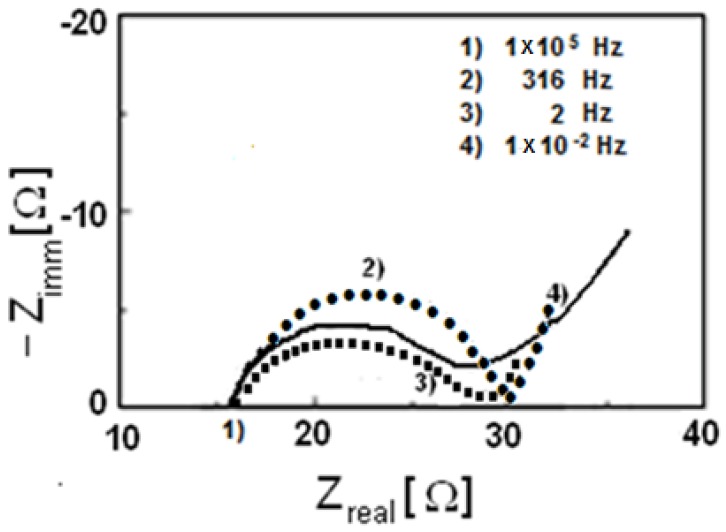
Impedance spectroscopy of: (**a**) • LiMn_0.1_Fe_0.9_PO_4_; (**b**) − LiMn_0.05_Fe_0.95_PO_4_; and (**c**) ▪ LiFePO_4_.

Impedance spectroscopy has been also performed on these three materials and in Figure 13 (Nyquist diagram) the real and imaginary parts of impedance have been reported.

The electrolytic resistance R_el_ is the first intercept of the semicircle with the *x*-axis, while the second intercept minus the electrolytic resistance R_el_ represents the charge transfer resistance R_ct_. Therefore, it is possible to state that for all cases R_ct_ is about 15 Ω. It is known by literature that the transfer charge resistance for pristine LiFePO_4_ without in situ carbon formation is higher than 40–50 Ω [33,34], so that by using this synthetic method a decrease of the charge transfer resistance has been, overall, reached.

Finally, we compared the synthesis and the electrochemical performances of our materials with analogous LiMn_x_Fe_(1−x)_PO_4_ already described in the literature and reported in Table 6.

Saravanan et al. [35] produced LiMn_x_Fe_(1−x)_PO_4_ /C (x = 0; 0.5; 1) with in situ carbon formation, by using the solvothermal method. They obtained a specific capacity equal to 150, 65, and 50 mAh/g for x equal to 0, 0.5 and 1, respectively, after 20 cycles at C/5. The same material obtained by Yoncheva et al. [36] at 500 °C starting from a phosphonate-formate precursor, freeze-drying an aqueous solution containing Li, Fe and Mn phosphate and formate ions, on the other hand, showed a capacity of 140, 120, and 95 mAh/g for x equal to 0, 0.5 and 1, respectively, a C/20. Zhang et al. [37] produced LiMn_x_Fe_(1−x)_PO_4_/C by solid state reaction with x = 0.7, 0.8, and 0.9, obtaining at C/10 a capacity ranging from 110 to 130 mAh/g as x decreases. Xu et al. [38] synthesized carbon free materials through a direct hydrothermal process a 170 °C achieving a capacity of 140, 110, 95, 90, and 78 mAh/g for x equal to 0.1, 0.2, 0.05, 0, and 0.4, respectively, at C/10. The lowest charge transfer resistance has been obtained for x = 0.1 and it is about 200 Ω, while, for x = 0.2 and x = 0, it is 450 and 1400 Ω, respectively.

Finally Wang et al. [39], which attained carbon-free LiMn_x_Fe_(1−x)_PO_4_ by mechano-activation assisted synthesis, reached, the best performance of 125 mAh/g for x equal to 0.2 at C/10.

On the basis of these considerations, our results are consistent and, in some cases, even better than those found in the literature.

## 4. Experimental

SEM analysis were obtained by the high-resolution microscope FE-SEM Auriga-Zeiss. The apparatus is also equipped with an EDX (Energy Dispersive X-Ray) detector (Bruker, Milan, Italy). Room temperature powder X-ray diffraction was performed by using Cu-Kα radiation λ = 0.15418 nm (Philips PW 1830 generator and Seifert XRD-3000 diffractometers). The data were collected with a step size of 0.02° and at count time of 4 s per step (0.3°·min^−1^) over the range 15° ≤ 2θ ≤ 80°. The powder diffraction pattern was indexed by using a Rietveld profile analysis [40].

Thermogravimetric (DSC-TGA) data of the precursor mixtures were obtained in flowing dry nitrogen at a heating rate of 10 °C·min^−1^ on a TA Instruments SDT Q600 thermogravimetric analysis. The FT-IR absorption spectra were recorded on a Shimadzu Prestige 21 FT-IR spectrophotometer using KBr pellets. BET (Fisons Instruments) analyses have been performed at liquid nitrogen temperature and using gaseous N_2_ to evaluate the specific surface of powders. Elemental analysis has been performed by the Servizio di Microanalisi del ISM-CNR, Monterotondo, Rome, Italy. Electrochemical characterization of samples was performed in T-shaped battery cells with lithium metal as counter (anode) and reference electrode. The cathode electrode contains about 10 mg of electroactive material with 10 wt % of Carbon Super S and 5 wt % of Teflon. The electrolyte is constituted by a glass wool separator filled with a 1 M solution of LiPF6 in 1/1 ethylene carbonate/diethyl carbonate. Potential step voltammetry (PSV) was carried out in a three-electrode cell configuration by using the following setting values: potential step: 0.02 V, relaxation time: 10 min, step duration: until I > Io/30 or 10 s if Io < 0.01 mA and in the range 3.2–3.7 V versus lithium. The same configuration was used for the potentiostatic–intermittent titration technique (PITT) experiments. The electrochemical impedance spectroscopy (EIS) has been performed in a frequency range from 10^5^ to 10^−2^ Hz, with a voltage amplitude of 0.01 V applied on a cell voltage of 3.47 V. A frequency response analyzer (Solartron 1255 HF and Solartron 1286 models by EG and G) and a galvanostat–potentiostat (Mac-Pile II Biologic) were used for these experiments.

## 5. Conclusions

Hybrid organic-inorganic precursors based on metal(II) phenylphosphonates have been synthesized, characterized and used for the synthesis of different LiFe_(1−x)_Mn_x_PO_4_ composites. First of all, LiMn_0.1_Fe_0.9_PO_4_ has been prepared following two different synthetic routes, i.e., using as organic precursors either Fe_0.9_Mn_0.1_(C_6_H_5_PO_3_)·H_2_O (**P1** precursor) or a mixture of Fe(C_6_H_5_PO_3_)·H_2_O and Mn(C_6_H_5_PO_3_)·H_2_O (**P2** and **P3** precursors). The materials thus obtained show similar behaviour, even if the sample prepared by using a mixture of **P2** and **P3** precursors presents a slightly higher capacity and seems to be more stable upon cycling. Subsequently, a series of LiMn_(1−x)_Fe_x_PO_4_ (with x = 0.05, 0.1, 0.5, 0.9 and 0.95) has been produced by using only **P2**, **P3**, and Li_2_CO_3_ precursors mixtures. Structural and morphological characterizations have been carried out analysing the effect of the reciprocal presence of iron and manganese on the electrochemical performances. Enhancing the manganese content, the capacity decreases remarkably and the only advantage is the presence of a second charge-discharge plateau with higher potential value. Moreover, the reversibility degree of the insertion-deinsertion process increases, the pseudo-diffusion lithium coefficient increases only slightly and the charge transfer resistance almost keeps constant, being in every cases quite lower than the corresponding values reported in literature for pristine LiFePO_4_. This is due to the presence of carbon produced in situ during the synthesis, which seems to be the only component able to increase substantially the electrochemical performances of this cathode material.

## Figures and Tables

**Table 1:** Molecular formulas of precursors.

Material	Formula
**P1**	Fe_0.9_Mn_0.1_(C_6_H_5_PO_3_)(H_2_O)
**P2**	Fe(C_6_H_5_PO_3_)(H_2_O)
**P3**	Mn(C_6_H_5_PO_3_)(H_2_O)

**Table 2:** Lattice parameters of precursors (in Å), where α = β = γ = 90°.

Material (Symbol)	a	b	c
Fe_0.9_Mn_0.1_(C_6_H_5_PO_3_)·H_2_O (**P1**)	5.680	14.410	4.900
Fe(C_6_H_5_PO_3_)·H_2_O (**P2**)	5.652	14.404	4.882
Mn(C_6_H_5_PO_3_)·H_2_O (**P3**)	5.751	14.401	4.953

**Table 3:** Cell parameters and crystallite size.

Compound	a (Å)	b (Å)	c (Å)	Crystallite Size T (Å)
LiFePO_4_	10.330	6.010	4.690	393
LiFe_0.95_Mn_0.05_PO_4_	10.335	6.011	4.693	370
LiFe_0.9_Mn_0.1_PO_4_	10.347	6.020	4.699	368
LiFe_0.5_Mn_0.5_PO_4_	10.372	6.054	4.706	365
LiFe_0.1_Mn_0.9_PO_4_	10.448	6.103	4.743	282
LiFe_0.05_Mn_0.95_PO_4_	10.448	6.104	4.743	280
LiMnPO_4_	10.450	6.108	4.732	265

**Table 4:** Specific surface of cathodic powder and carbon percentage.

Material (Symbol)	Specific Surface Area (m^2^·g^−1^)	Carbon Content (%)
LiFePO_4_	105	10.0
LiMn_0.05_Fe_0.95_PO_4_	100	10.2
LiMn_0.1_Fe_0.9_PO_4_	130	12.5
LiMn_0.5_Fe_0.5_PO_4_	115	10.3
LiMn_0.9_Fe_0.1_PO_4_	110	11.3
LiMn_0.95_Fe_0.05_PO_4_	151	13.7
LiMnPO_4_	105	11.5

**Table 5:** Pseudo-diffusion coefficients D (in cm^2^·s^−1^).

Material (Symbol)	D (cm^2^·s^−1^)
LiFePO_4_	2.0 × 10^−14^
LiMn_0.05_Fe_0.95_PO_4_	5.7 × 10^−14^
LiMn_0.1_Fe_0.9_PO_4_	7.7 × 10^−14^

**Table 6:** Electrochemical performances of analogous LiMn_x_Fe_(1−x)_PO_4_ material described in the literature.

Compound	Method	C-Rate	Capacity (mAh/g)	Reference
LiMn_x_Fe_(1−x)_PO_4_/C (x = 0; 0.5; 1)	Solvothermal process	C/5	150; 65; 50	[35]
LiMn_x_Fe_(1−x)_PO_4_/C (x = 0; 0.5; 1)	freeze-dry process	C/20	140; 120; 95	[36]
LiMn_x_Fe_(1−x)_PO_4_/C (x = 0.7; 0.8; 0.9)	Solid state reaction	C/10	110; 120; 130	[37]
LiMn_x_Fe_(1−x)_PO_4_ (x = 0; 0.05; 0.1; 0.2; 0.4)	Hydrothermal process	C/10	140; 110; 95; 90; 78	[38]
LiMn_x_Fe_(1−x)_PO_4_ (x = 0; 0.1; 0.2; 0.3)	Mechano-activation synthesis	C/10	135; 108; 125; 80	[39]
